# Use of Nalmefene in Routine Practice: Results from a French Prospective Cohort Study and a National Database Analysis

**DOI:** 10.1093/alcalc/agab029

**Published:** 2021-05-10

**Authors:** Henri-Jean Aubin, Caroline Dureau-Pournin, Bruno Falissard, François Paille, Alain Rigaud, Sophie Micon, Marine Pénichon, Frank Andersohn, Christine Truchi, Patrick Blin

**Affiliations:** Université Paris-Saclay, Inserm, CESP, AP-HP. Université Paris Saclay, 12, avenue Paul-Vaillant-Couturier, 94804 Villejuif, France; Université de Bordeaux, INSERM CIC-P 1401, Bordeaux PharmacoEpi, Rue Eugène Jacquet, Bordeaux 33000, France; CESP/INSERM U1018, Centre de Recherche en Epidémiologie et Santé des Populations, Hôpital Paul Brousse Bat 15/16, 16 av PV Couturier - 94807 Villejuif, France; Université de Lorraine, 34 Cours Léopold, 54000 Nancy, France; Ancien chef du pôle d'Addictologie de l'EPSM Marne, Psychiatre des hôpitaux honoraire, 28 bis, rue de Courcelles 51100 Reims, France; Université de Bordeaux, INSERM CIC-P 1401, Bordeaux PharmacoEpi, Rue Eugène Jacquet, Bordeaux 33000, France; Université de Bordeaux, INSERM CIC-P 1401, Bordeaux PharmacoEpi, Rue Eugène Jacquet, Bordeaux 33000, France; Frank Andersohn Consulting and Research Services, Mandelstrasse 16, 10409 Berlin, Germany; Lundbeck SAS, 102 Terrasse Boieldieu 92085 Paris La Défense, France; Université de Bordeaux, INSERM CIC-P 1401, Bordeaux PharmacoEpi, Rue Eugène Jacquet, Bordeaux 33000, France

## Abstract

**Aims:**

Two complementary studies were used to assess the real-life use of nalmefene in alcohol-dependent patients and its impact on alcohol use health status.

**Methods:**

USE-PACT was a prospective cohort study designed to evaluate the real-life effectiveness of nalmefene in the management of alcohol dependence, as assessed by total alcohol consumption (TAC) and number of heavy drinking days (HDD) at 1 year. USE-AM was a cohort study using data from the French nationwide claims database and was used to evaluate the external validity of the population in the prospective study.

**Results:**

Overall, 256 of 700 new nalmefene users enrolled in the USE-PACT study had valid data at 1 year. After 1 year, patients treated with nalmefene showed a mean ± SD reduction from baseline in TAC (−41.5 ± 57.4 g/day) and number of HDD (−10.7 ± 11.7 days/4 weeks). Patients took a mean ± SD of 20.0 ± 12.0 tablets/4 weeks (median of 1 tablet/day) for the first 3 months and then reduced the dose. The proportion of patients who no longer took nalmefene gradually increased from 5% at 1 month to 52% at 1 year. The USE-AM study identified 486 patients with a first reimbursement for nalmefene in 2016; baseline characteristics confirmed external validity of the USE-PACT study. Overall, 46.3% of initial USE-AM prescriptions were made by GPs; most (91.8%) patients stopped treatment during follow-up. However, 15.2% of patients resumed treatment after stopping.

**Conclusions:**

In this analysis of French routine practice, patients with alcohol dependence treated with nalmefene showed reduced alcohol consumption, and nalmefene was generally well tolerated.

## Teaser

**Short Summary:** Two studies were used to evaluate how nalmefene is used in the management of alcohol dependence in France. After 1 year, new nalmefene users had substantially reduced alcohol consumption. Insurance data confirmed external validity of the population sample and the observation that patients often discontinue treatment over 1 year.

## Introduction

Per capita, alcohol consumption in France remains amongst the highest in the world, although it has been steadily decreasing in recent years (WHO, 2018). Dose-dependent relationships have been shown for alcohol consumption and the risks of liver disease, cardiovascular disease, cancer and suicide (WHO, 2018, 2019), with an exponential relationship between alcohol use and the risk of death (Rehm *et al.,* 2001). Over time, consistent unhealthy drinking may lead to physiological changes in regions of the brain and the development of alcohol dependence (Levey *et al.,* 2014; WHO, 2018; Carvalho *et al.,* 2019).

Despite the associated high level of disease burden, it has been estimated that only 10% of European patients with alcohol dependence receive the treatment they need (Rehm *et al.,* 2013; Mann *et al.,* 2017). It has been argued that offering a treatment strategy able to better meet the patients’ preferences and needs may reduce this treatment gap by increasing treatment seeking in people with an alcohol use disorder (Luquiens and Aubin, 2014). More broadly, new avenues of success are to be found in the development of personalized treatment approaches aiming at improving patient matching with medications (Litten *et al.,* 2015; Witkiewitz *et al.,* 2019). In 2015, the French Alcohol Society updated their guidelines to reflect the needs of the modern day French addiction care system. The revised guidelines recognized that total abstinence may not be an achievable aim for many patients and recommended that reduction of alcohol consumption can be considered as an alternative approach to reducing negative consequences in people with alcohol dependence (Rolland *et al.,* 2016).

Nalmefene (Selincro^®^, H. Lundbeck A/S, Valby, Denmark) is an opioid system modulator licensed in Europe and Japan for the reduction of alcohol consumption in adult patients with alcohol dependence who have a high drinking risk level (high DRL is defined as alcohol consumption >60 g/day for men and >40 g/day for women), without physical withdrawal symptoms and who do not require immediate detoxification. It was the first drug to receive regulatory approval for the indication of reducing drinking in alcohol-dependent individuals and the updated French guidelines recommended nalmefene as the first-line medication for reducing alcohol consumption in people with alcohol dependence (grade A) (Rolland *et al.,* 2016). Regulatory approval of nalmefene in Europe was based on the results of phase III clinical trials, which consistently demonstrated that nalmefene, given to alcohol dependent patients with a high DRL, on an as-needed basis and together with psychosocial support, reduces the total amount of alcohol consumption (TAC) and number of heavy drinking days (HDDs) (Gual *et al.,* 2013; Mann *et al.,* 2013; van den Brink *et al.,* 2013, 2014). However, following European approval, additional data were required by the French Health Technology Assessment agency (HAS, Haute Autorité de Santé) to inform on the real-world effectiveness and safety of nalmefene as given in routine practice in France.

We aimed to document the ‘real-life’ use of nalmefene and evaluate its impact on alcohol use health status. Two different studies were performed to address these aims:

1. A prospective, multi-centre cohort study (**U**se of **Se**lincro^®^ and im**pact** in routine practice, USE-PACT study)
2. A historical cohort study using data from the French National Health Insurance database (USE-AM).

The advantage of the dual-study approach is that the inherent limitations of one study design can be addressed with the strengths of the other one. For example, the national database study can be used to evaluate the external validity and generalizability of the patient population enrolled in the prospective cohort study.

## Methods

### USE-PACT study design

USE-PACT was a prospective national cohort study to assess the real-life use of nalmefene and its impact on alcohol consumption at 1 year. It was registered with the clinicaltrial.gov registry (NCT02492581) and EU PAS registry (EUPAS11854) and was conducted in accordance with European Network of Centres for Pharmacoepidemiology and Pharmacovigilance (ENCePP) guidance endorsed by the European Medicines Agency on best practices for conducting and reporting post-authorization safety studies. The protocol received all legal approvals and authorizations from the national medical committee (Conseil National de l’Ordre des Médecins, CNOM), the data-protection committee for biomedical research (Comité Consultatif sur le Traitement de l’Information en matière de Recherche dans le domaine de la Santé, CCTIRS) and the national data-protection authority (Commission Nationale de l’Informatique et des Libertés, CNIL).

The study was conducted in a random sample of French prescribers. The prescribing physician sample included general practitioners, psychiatrists and physicians practicing within an addiction specialist setting. The main inclusion criteria required patients to be consenting adults ≥18 years who were starting treatment with nalmefene. Patients should not have been included in another study likely to modify their management, and they should not have been under guardianship.

Data were collected via standardized paper questionnaires completed by the investigators and patients. At baseline, the physicians had to complete an inclusion questionnaire documenting: general patient characteristics, disease history, alcohol consumption as assessed by TAC, number of HDDs and DRL according to the WHO classification (WHO, 2000; EMA, 2010). In addition, physicians provided a review of the consultation, including alcohol consumption target, associated treatments and date of the next consultation. Patients attended visits at 1, 3, 6, 9 and 12 months, and at each visit, physicians completed follow-up questionnaires documenting nalmefene treatment, consultations that had taken place since the last visit, laboratory workup, clinical global impression of severity (CGI-S) (Forkmann *et al.,* 2011), alcohol consumption. When liver function tests were recorded, analyzed variables included γ-glutamyltransferase (GGT), alanine aminotransferase (ALT) and aspartate aminotransferase (AST). Investigators also documented any adverse events.

Patients also completed a series of self-assessment questionnaires at each visit, including patient-rated quality of the psychosocial follow-up and global impression (CGI-P) (Forkmann *et al.,* 2011). Impact on quality of life was evaluated with the Alcohol Quality of Life Scale (AQoLS) (Luquiens *et al.,* 2016) and EuroQol questionnaire (EQ-5D-5L) (EuroQol, 1990), and functional impact was evaluated using the Sheehan Disability Scale (SDS) (Sheehan *et al.,* 1996). Best efforts were made to collect data on patients who were ‘lost to follow-up at 12 months’, firstly via a telephone interview with the patient or, failing that, with their attending physician.

For the USE-PACT study, we estimated that a sample size of 600 patients was needed to obtain precision of ±5% for the relative variation in TAC, with a standard deviation of 63% and 95% confidence intervals. Assuming a dropout rate of 40%, a total of 1000 patients were required for recruitment. All statistical analyses performed were descriptive in nature. In order to understand the impact of treatment in different treatment groups, we also assessed results of TAC and HDDs by DRL at baseline.

### USE-AM study design

USE-AM was performed as a cohort study in a healthcare reimbursement database based on data from the National Health Data System (SNDS) in France. The SNDS database links claims, with hospital-discharge summaries (PMSI) and the national death registry, using pseudonymization of the unique national identifier. It currently includes 98.8% of the French population (i.e. more than 66 million people from birth/immigration) until their death/emigration) (Bezin *et al.,* 2017). The Echantillon Généraliste de Bénéficiaires (EGB) is a permanent representative sample of the SNDS, which includes currently about 700,000 beneficiaries whether or not they have received reimbursement of care. It is representative of the entire French population, in terms of sex, age and average expense reimbursed per consumer (Studer *et al.,* 2009). Access to SNDS was approved by the national data protection authority (CNIL) after advice from a committee on healthcare data research (CEREES).

The USE-AM study included adult (≥18 years old) patients from the EGB sample who have had a first dispensing of nalmefene over a period of 1 year, between the 1 January and 31 December 2016. Patients should have had ≥2 years of history in the database, and ≥1 year of follow-up (i.e. coverage in the database). The time of follow-up was set at 1 year after index date (day of first nalmefene dispensing). For each patient in the study population, the following data were extracted: EGB affiliation history, socio-demographic data, long-term medical condition data occurring up to the index date (along with ICD-10 codes, and start and end date); drug dispensations between 1 January 2013 and 31 December 2016; consultations and medical visits carried out between 1 January 2013 and 31 December 2016 (completion date, physician specialty). All statistical analyses performed were descriptive in nature.

## Results

### USE-PACT results

**Fig. 1.**
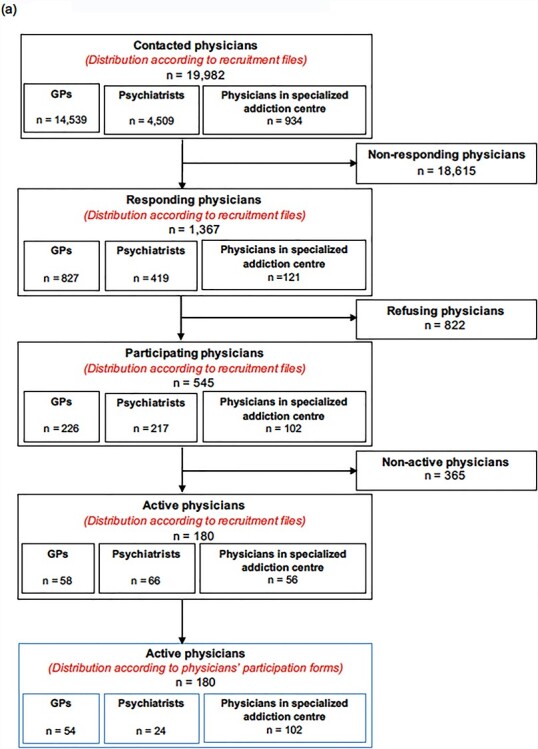

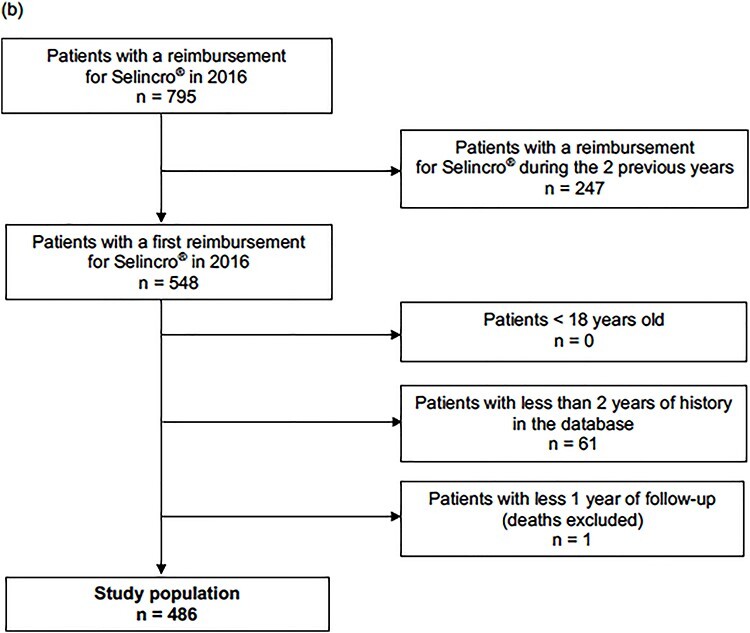
Study flow (**a**) USE-PACT (**b**) USE-AM.

A total of 19,982 physicians were contacted by mail between January and April 2016 to participate in the USE-PACT study. Of these, 545 physicians agreed to participate and 180 included at least one patient in the study. Overall, 54 GPs, 24 psychiatrists and 102 physicians working in specialized addiction centres included ≥1 patient in the study (Figure 1a). Among the HCPs (*n* = 822) who explained their refusal to participate, the most common reasons were lack of eligible patients (48.3%) and lack of time (36.1%).

Between 23 February 2016 and 31 December 2016, 700 patients were included in the study (safety data set), 256 of whom had valid data at 12 months of follow-up and were included in the analyses of effectiveness (effectiveness set). Baseline data for both analysis sets are provided in Table 1. About two-thirds of patients were men, the median age was 48 years and about 60% had been aged less than 35 years alcohol consumption problem onset. The mean ± SD TAC was 64.9 ± 55.8 g/day, mean HDD was 16.2 ± 10.5 days per 4 weeks and half the patients (50.8%) had a high or very high DRL. About half of the patients (51.2%) had previously received prior pharmacological treatment for alcohol dependence, the median being one treatment, and the majority had at least one psychiatric comorbidity. As recommended in the prescribing information, the majority of the 256 patients had regular follow-up visits with their physician in charge of their alcohol use disorder (74.6–83.6% at each follow-up point). Participating physicians provided psychosocial support of their patients throughout the follow-up period, with quality of communication regarding the consumption of alcohol and its impact evaluated as sufficient by the patients during the follow-up period (*n* = 79 patients returned the follow-up self-assessment questionnaires at 12 months).

**Table 1:** Baseline characteristics

Characteristic	USE-PACT		USE-AM
	Safety set (*N* = 700)	Efficacy set (*N* = 256)	(*N* = 486)
Sex (male/female); *n* (%)	471 (67.3)/229 (32.7)	167 (65.2)/89 (34.8)	338 (69.5)/148 (30.5)
Age (years)			
Mean ± SD Median [p25%, p75%]	48.3 ± 10.8 48 [42, 56]	48.9 ± 10.9 48 [42, 57]	49.5 ± 11.6 50 [41, 57]
Age of onset of alcohol consumption problems; *n* (%)			
<18 years 18–24 years 25–34 years 35–44 years 45–54 years 55–64 years ≥65 years	124 (17.7) 166 (23.7) 162 (23.1) 144 (20.6) 73 (10.4) 26 (3.7) 3 (0.4)	49 (19.1) 56 (21.9) 49 (19.1) 60 (23.4) 29 (11.3) 13 (5.1) 0 (0.0)	N/A
Number patients with ≥1 psychiatric co-morbidity, *n* (%)^a^	392 (56.0)	162 (63.3)	111 (22.8)
Depression Anxiety Bipolar disorder	235 (33.6) 129 (18.4) 42 (6.0)	97 (37.9) 54 (21.1) 23 (9.0)	41 (8.4) 5 (1.0) 19 (3.9)
Total alcohol consumption (g/day)			
Mean ± SD Median [p25%, p75%]	66.0 ± 57.9 54.3 [27.1; 85.7]	64.9 ± 55.8 52.7 [28.6; 89.3]	N/A
Number of heavy drinking days (per 4 weeks) Mean ± SD Median [p25%, p75%]	16.1 ± 10.2 18 [6; 28]	16.2 ± 10.5 17 [6; 28]	N/A
Current concomitant medications; *n* (%)			
Any current treatment Gastrointestinal system/metabolism Cardiovascular system Central nervous System Psycholeptic Psychoanaleptic Antiepileptic Addiction Analgesic	544 (77.7) 124 (17.7) 97 (13.9) 488 (69.7) 415 (59.3) 280 (40.0) 64 (9.1) 21 (3.0) 18 (2.6)	208 (81.3) 45 (17.6) 36 (14.1) 188 (73.4) 164 (64.1) 118 (46.1) 36 (14.1) 8 (3.1) 9 (3.5)	358 (73.7) 87 (17.9) 85 (17.5) 303 (62.3) 249 (51.2) 147 (30.2) 24 (4.9) 12 (2.5) 49 (10.1)
History of prior pharmacological treatment for alcohol, *n* (%) Acamprosate Baclofen Naltrexone Disulfiram	362 (51.7) 242 (34.6) 134 (19.1) 111 (15.9) 59 (8.4)	131 (51.2) 85 (33.2) 47 (18.4) 37 (14.5) 27 (10.5)	146 (30.0) 69 (14.2) 71 (14.6) 36 (7.4) 12 (2.5)
History of prior treatment goal, *n* (%)			
Abstinence Abstinence and reduction Reduction	221 (31.6) 215 (30.7) 169 (24.1)	83 (32.4) 78 (30.5) 55 (21.5)	N/A

^a^Comorbidities in USE-AM were limited to those classified as long-term disease registrations

Patients took a mean of 20.0 ± 12.0 nalmefene tablets per 4 weeks (median of 1 tablet/day) for the first 3 months then reduced the dose to reach a mean of 14.2 ± 13.2 tablets at 9 months and 12.1 ± 13.4 tablets per 4 weeks at 12 months. The proportion of patients who no longer took nalmefene gradually increased from 5% at 1 month to 52% at 12 months. Reasons for stopping treatment with nalmefene were diverse: clinical improvement (from 2% of patients at 1 month to 15% at 12 months), adverse events (between 10 and 15% at each follow-up time) and lack of efficacy (from 2% at 1 month to 11% at 12 months).

**Fig. 2.**
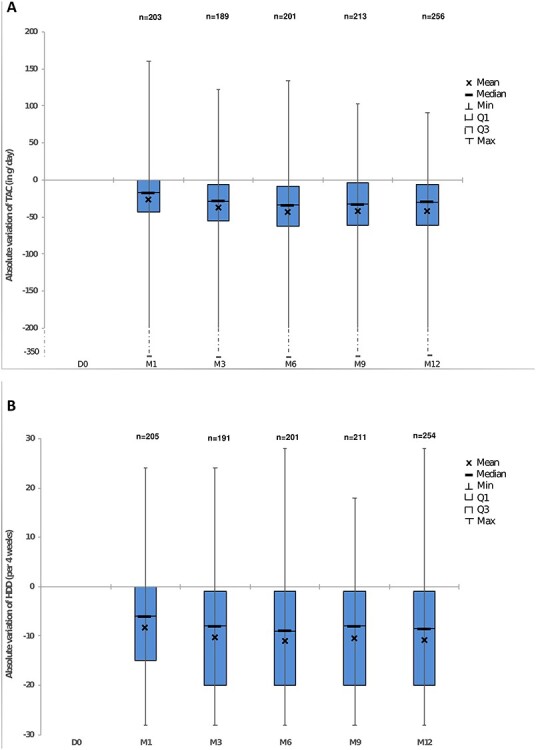
Evolution of alcohol consumption over 12 months in USE-PACT (**a**) TAC (**b**) number of HDD.

**Fig. 3.**
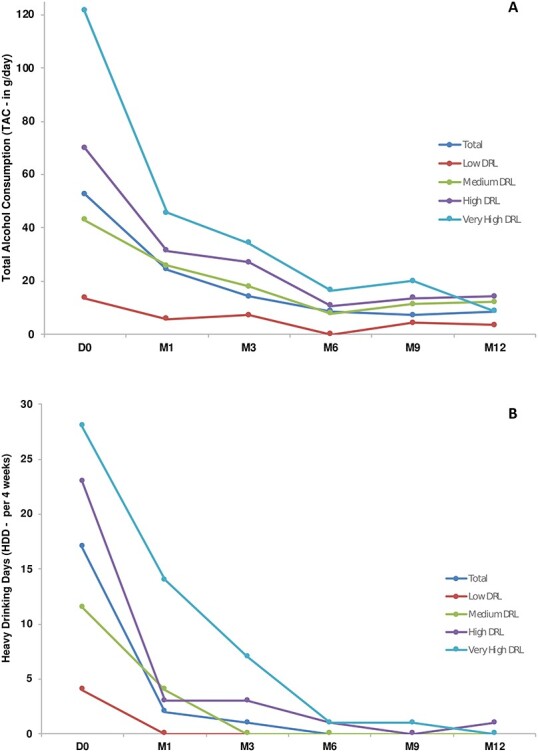
Reduction in (**a**) total alcohol consumption (TAC) and (**b**) number of heavy drinking days (HDD) by baseline drinking risk level in USE-PACT.

A reduction in TAC and number of HDD was observed at 1 year. This reduction was apparent from Month 1 onwards (first follow-up visit) and reductions continued for the first 6 months before stabilizing until study end. As shown in Figure 2, mean TAC at 1 year had reduced from baseline by −41.5 ± 57.4 g/day and mean HDDs had reduced from baseline by −10.7 ± 11.7 days/4 weeks. Effect sizes (Cohen, 1988) of the mean change from baseline to Month 12 were substantial for both TAC (*d* = −0.72) and HDD (*d* = −0.91). Overall, 68.8% of patients decreased their TAC over the 12-month period, and 71.1% of patients had a decrease in number of HDD. The reduction in alcohol consumption was reflected in shifts from high to lower DRL levels over 12 months. The proportion of patients at high/very high DRL reduced from 50.8% at baseline to 14.9% of patients at 12 months of follow-up. Subgroup analysis restricted to patients with high or very high DRL at baseline showed greater improvement in TAC and HDD (Figure 3). After 12 months over half (54.3%) of patients were considered to have achieved their goal of reduced alcohol consumption with a further 26.6% of patients considered as partially achieving their goal, and the mean TAC and HDD was 23.3 ± 35.2 g/day and 5.4 ± 9.2 days/4 weeks, respectively.

Where available, results of liver function tests showed slight reductions (improvements) following treatment. Median levels of GGT reduced from 85 IU/L (*n* = 93) at baseline to 48.5 IU/l at 12 months (*n* = 64). Similarly, median AST levels reduced from 36.5 IU/l at baseline (*n* = 84) to 31 IU/l at 12 months (*n* = 57) and ALT levels reduced from 39.0 IU/l (*n* = 85) at baseline to 34 IU/l at 12 months (*n* = 57). Although the patient-reported data were limited by the low rate of return of completed self-assessment questionnaires (*n* = 79/256 at 12 months), patients generally self-reported improvements in CGI-P (78.5% of patients reported improvement at 12 months). The median SDS score decreased from 14.0 at baseline (*n* = 172) to 2.0 at 12 months (*n* = 58) and the median AQoLS scale decreased from 7.0 at baseline (*n* = 238) to 3.0 at 12 months (*n* = 78). In addition, there was an increase in the median score for generic quality of life (EQ-5D- 5 L). EQ-5D- 5 L scores increased from 0.77 at baseline to 0.93 at 12 months and the number of patients with a score ≥ 0.8 increased from 36.7% (*n* = 94/234) at baseline to 70.9% (*n* = 56/78) of patients at 12 months.

Overall, 396 adverse events were reported in 146 patients (20.9%). The most commonly reported adverse events were nausea (4.7%), dizziness (2.7%) and insomnia (2.4%) (Table 2). A total of 85 events observed in 40 patients (5.7%) were considered serious. Twelve patients (1.7%) died between inclusion and the 12-month follow-up point: 5 from causes not related to treatment (suicide in the context of a family conflict, road accident [hit by a car when walking], mesenteric ischemia following surgery for pleural lung disease, seizure and probable pulmonary embolism), and 7 for which a relationship to nalmefene treatment was not assessed by the investigator or assessed as unknown (due to loss-to-follow up after the last assessment). No patient had an event of alcohol withdrawal syndrome during the study.

**Table 2:** Safety reporting in the USE-PACT study

Safety parameter	Safety set (*N* = 700)	Efficacy set (*N* = 256)
≥1 AE	146 (20.9)	46 (18.0)
≥1 treatment-related AE	129 (18.4)	44 (17.2)
AE (preferred term) reported in ≥1 patient		
Nausea	33 (4.7)	7 (2.7)
Feeling dizzy	19 (2.7)	9 (3.5)
Insomnia	17 (2.4)	7 (2.7)
Drug ineffectiveness	14 (2.0)	4 (1.6)
Malaise	13 (1.9)	3 (1.2)
Vertigo	12 (1.7)	2 (0.8)
Hyperhidrosis	11 (1.6)	7 (2.7)
Tremor	11 (1.6)	4 (1.6)
Vomiting	10 (1.4)	2 (0.8)
Headache	9 (1.3)	3 (1.2)
Asthenia	9 (1.3)	2 (0.8)
Drowsiness	8 (1.1)	1 (0.4)
Anxiety	8 (1.1)	1 (0.4)
Fatigue	7 (1.0)	2 (0.8)
Abnormal sensation	7 (1.0)	2 (0.8)
Visual hallucination	4 (0.6)	1 (0.4)
Hallucination	3 (0.4)	0 (0.0)

### USE-AM results

A total of 486 patients were identified with a first reimbursement for nalmefene (Selincro^®^) between 1 January 2016 and 31 December 2016. Baseline characteristics were similar to those of the patients included in the USE-PACT study (Table 1). A majority were men (69.5%) with a mean age of 49.5 ± 11.6 years. The USE-AM study only considered comorbidities based on long-term disease status (*‘Affection de Longue Durée’*), and thus the percentage of patients with a psychiatric comorbidity appeared lower than that in the USE-PACT study (22.8 vs 56.0%). On the other hand, current prescription rates for central nervous system (CNS) medications were similar between the two studies.

The majority of initial prescriptions of nalmefene treatment were made by GPs (46.3% of patients), followed by physicians of unknown specialty practicing in a hospital facility (30.9% of patients), and psychiatrists (14.6% of patients). The median number of packs dispensed at the time treatment was initiated was two packs per patient, i.e. a minimum of 1 month of treatment (based on 1 tablet/day). Virtually, all (91.8%) patients stopped treatment (no dispensing of nalmefene for at least 3 months) during 1 year follow-up, with almost half of patients (48.4%) having received a single dispensed treatment and one in five (19.5%) patients having received two dispensed treatments. However, more than one in 10 patients (15.2%) ultimately resumed treatment after first stopping. A total of 10 patients in USE-AM died during the 1 year follow-up (2.1%), but the majority of these (7 out of 10) occurred ≥3 months after the last treatment was dispensed, making a relationship with the product unlikely.

## Discussion

Results of these two complementary post-authorization studies provide a ‘real-world’ perspective from France on the findings previously reported in phase III pivotal studies (Gual *et al.,* 2013; Mann *et al.,* 2013; van den Brink *et al.,* 2014; Miyata *et al.,* 2019) as well phase IV studies conducted in European primary care (Castera *et al.,* 2018) and in Spain (Barrio *et al.,* 2019). Patients enrolled in the USE-PACT study showed clear reductions in TAC and number of HDD at 1 year, and effect sizes were substantial for both measures (effect sizes of −0.72 and *d* = −0.91, for TAC and HDD, respectively). Reductions in alcohol consumption were apparent after 1 month and continued for the first 6 months. Treatment was generally well tolerated, adverse events showing a profile similar to that observed in clinical trials (Gual *et al.,* 2013; Mann *et al.,* 2013; van den Brink *et al.,* 2014).

In the USE-PACT study, the reductions in TAC and HDDs followed a similar time course as seen in the pivotal studies (Gual *et al.,* 2013, Mann *et al.,* 2013, van den Brink *et al.,* 2014), and in particular, the ESENSE 2 trial, which included patients from France (Gual *et al.,* 2013). While baseline alcohol consumption was somewhat lower than in the pivotal studies (65 vs. 92 g/day in ESENSE 2), we observed a similar magnitude of reduction (~64% in USE-PACT vs. ~68% reduction in TAC in the ESENSE 2 nalmefene group). Also consistent with clinical trial results, the reduction in TAC and HDDs at 6 months and at 1 year was more pronounced in patients with a high/very high heavy drinking risk on starting nalmefene (van den Brink *et al.,* 2013). This corresponds to the approved indication of nalmefene use. While it appears that almost half of patients had a medium or low risk DRL at baseline (nalmefene is indicated for patients with high DRL), this discrepancy may be partly explained by the fact that USE-PACT patients had already come in to see their physician for treatment and may already have reduced their consumption from earlier levels. In the ESENSE 2 trial, 33% of patients had reduced their drinking prior to start of treatment, before any intervention (Gual *et al.,* 2013).

Prescription patterns for nalmefene showed that many patients discontinue treatment over the course of 1 year. In particular, a significant proportion of patients in the USE-PACT study discontinued after only a couple of months—while remaining in the study follow-up. It is the authors’ clinical experience that there are several potential reasons for discontinuation. The early experience of adverse events, such as dizziness or nausea, which are usually mild to moderate and transient (van den Brink *et al.,* 2014), can be off-putting to the unprepared patient and prevent them from persevering with treatment. A small proportion of patients weary of taking any drug after some time, and some patients, although wishing to reduce (but not stop) their drinking report that they avoid the treatment because it prevents them to the pleasurable experience of intoxication. Finally, nalmefene is to be taken ‘as needed’, which may make it easier to forget versus a specific schedule for patients to follow. In USE-AM, where virtually all patients stopped treatment during 1 year of follow-up, the initial prescription of nalmefene was more commonly made by GPs, followed by doctors practising in hospital facilities (of unknown specialty) and psychiatrists. The longer use in the USE-PACT study (48% of patients were still on treatment at 12 months) may be due to (a) the higher proportion of patients treated in a specialist setting and (b) a greater incentive to remain on treatment during a formal, albeit observational, study.

The safety and tolerability profile of nalmefene was in line with previous trial reports and known profile (Gual *et al.,* 2013; Mann *et al.,* 2013; van den Brink *et al.,* 2014, 2015), and no new safety concerns were identified. The most commonly reported adverse events were nausea, dizziness, and insomnia, which are expected with opioid antagonism and were predominantly mild to moderate and transient. As discussed above, it is useful for patients to know that nausea and dizziness tend to occur within 1 day after the first dose and are generally of short duration (around 3 days) (van den Brink *et al.,* 2015). In the pivotal studies, recurrence of these frequent events was not related to the pattern of study medication intake, and there was no difference in safety for the patients when nalmefene was taken daily or intermittently (van den Brink *et al.,* 2015). In these real-world studies, rates of death were slightly higher than seen in the pivotal trials, but were in line with overall mortality rates in alcohol-dependent patients (Duberq *et al.,* 2020). The deaths were not considered related to nalmefene treatment in this study; there was one suicide recorded in the USE-PACT study, which was in the context of an emotionally unstable patient during a family conflict while intoxicated with alcohol. In the pivotal trials, there were no completed suicides in patients receiving nalmefene, but two patients receiving placebo committed suicide (van den Brink *et al.,* 2015).

In real-world studies, it is always a matter of discussion if participating practices and/or the included patients are truly representative for the patients actually treated in the respective health care system, or if selection bias may have influenced the results. Our dual-study approach allowed the comparison of the USE-PACT study with national healthcare data in the USE-AM study that is, by definition, not subject to selection bias. Patient characteristics were similar in both studies, thereby supporting the external validity of the prospective study. In the USE-AM study, the proportion of prescribers practicing in a hospital facility were lower compared to the USE-PACT study, which may also explain the slightly higher proportion of patients with complex co-morbidities and prior pharmacological treatment for alcohol in the USE-PACT study. However, coding systems were not the same for the two studies. In the USE-AM study, coding for type of physician was performed (by the dispensing pharmacists) according to the prescription, which could lead to doctors from a specialist facility being classified in the ‘hospital doctor (specialty unknown)’ group, or as general practitioners. In the USE-PACT study, the coding is based on the statement made by the doctor in the participation form. Likewise, comorbidities in USE-AM were limited to those classified as long-term disease registrations and it is notable that rates of current concomitant medications were more similar between the two studies. It should be noted, however, that our findings are somewhat specific to the French healthcare system, and therefore may not be fully generalizable in other countries. In particular, nalmefene (like other approved medications for alcohol use disorder) is reimbursed in France.

Although limited by missing data, results of the liver function tests, and patient reported measures of global function and quality of life show the relevance of reduced alcohol consumption in real-life practice. Other limitations included the lack of a control group and those inherent to observational studies such as the high loss to follow-up despite best attempts to keep these patients in the study. Unfortunately, difficulties in recruitment prevented us from reaching the target 1000 patients in the USE-PACT study. Since the prospective study was an uncontrolled observational trial, it is not possible to infer the role of nalmefene in patients’ improvements. Other factors such as therapeutic settings, psychosocial support, patient’s expectancies and regression to the mean (Rutherford and Roose, 2013) are likely to have impacted our observations. One should also keep in mind that, in this real-world study, a number of patients take other medications—for their alcohol use disorder as for comorbidities—that can interact with nalmefene in various ways. However, nalmefene is considered a relatively safe drug for co-administration, with the exception of opioid agonists (Guerzoni *et al.,* 2018).

This real-world study included patients being treated as per routine practice, and we do not know if patients had previously followed a detoxification treatment. However, nalmefene should not be used in patients requiring immediate detoxification (Selincro SmPC, 2018). While nalmefene is the only medication specially developed for alcohol reduction (as opposed to relapse prevention), naltrexone remains a possible alternative, as it has shown some efficacy in reducing alcohol drinking in heavy drinkers (Kranzler *et al.,* 2009). An indirect comparison meta-analysis of these two drugs concluded that nalmefene may be more effective than naltrexone (Soyka *et al.,* 2016), although whether a clinically relevant difference between the two medications really exists is still an open question. Side effects of nalmefene seem similar to naltrexone (Witkiewitz *et al.,* 2019).

In summary, in this routine practice study in France, patients with alcohol dependence treated with nalmefene, taken as needed, showed reduced alcohol consumption, and nalmefene was generally well tolerated. Substantial proportions of patients discontinued treatment over the 1-year follow-up periods. Nevertheless, the favourable results observed in USE-PACT in terms of alcohol consumption indicate that the actual level of nalmefene exposure in the study was sufficient to reach the observed effect in terms of drinking level reduction. These data form a vital part of the nalmefene post-launch risk management plan and provide important information for health authorities and payers.

## Author contributions

All authors contributed to the interpretation of the data, provided critical review of the manuscript and approved the manuscript for submission. Henri Jean Aubin, Frank Andersohn and Christine Truchi contributed equally to the first draft of the manuscript. Henri-Jean Aubin, Bruno Falissard, François Liard, François Paille and Alain Rigaud contributed to data acquisition. Patrick Blin and Caroline Dureau-Pournin contributed to the research design and supervised the study conduct, data analyses and report’s writing for the entire research. Sophie Micon managed the study and wrote the study report, and Marine Pénichon performed the data analysis.

## Data availability statement

The USE-PACT protocol and dataset is available from the corresponding author on reasonable request. USE-AM data are not available under French Law.
