# Corrigendum to: Use of Nalmefene in Routine Practice: Results from a French Prospective Cohort Study and a National Database Analysis

**DOI:** 10.1093/alcalc/agab046

**Published:** 2021-06-08

**Authors:** Henri-Jean Aubin, Caroline Dureau-Pournin, Bruno Falissard, François Paille, Alain Rigaud, Sophie Micon, Marine Pénichon, Frank Andersohn, Christine Truchi, Patrick Blin

**Affiliations:** 1 Université Paris-Saclay, Inserm, CESP, AP-HP. Université Paris Saclay, 12, avenue Paul-Vaillant-Couturier, 94804 Villejuif, France; 2 Université de Bordeaux, INSERM CIC-P 1401, Bordeaux PharmacoEpi, Rue Eugène Jacquet, Bordeaux 33000, France; 3 CESP/INSERM U1018, Centre de Recherche en Epidémiologie et Santé des Populations, Hôpital Paul Brousse Bat 15/16, 16 av PV Couturier - 94807 Villejuif, France; 4 Université de Lorraine, 34 Cours Léopold, 54000 Nancy, France; 5 Ancien chef du pôle d'Addictologie de l'EPSM Marne, Psychiatre des hôpitaux honoraire, 28 bis, rue de Courcelles 51100 Reims, France; 6 Frank Andersohn Consulting and Research Services, Mandelstrasse 16, 10409 Berlin, Germany; 7 Lundbeck SAS, 102 Terrasse Boieldieu 92085 Paris La Défense, France

When this paper first published, it was missing the below conflict of interest statements from the authors. These conflict of interest statements now appear in the paper online.

Dr Patrick Blin wishes to record that he is an employee of the Bordeaux PharmacoEpi team in the University of Bordeaux, and the studies were performed by this team at the request of the French Health Technology Assessment agency, with a grant from Lundbeck and according to the European Network of Centres for Pharmacoepidemiology and Pharmacovigilance (ENCEEP) code of conduct and independence.

Dr François Paille wishes to record that he has received payments for consulting activity (Lundbeck, Indivior, D&A Pharma, and Kinnov therapeutics) and for invitations to speak at conferences connected with Lundbeck, Indivior and Pfizer.

